# Perceptions of perioperative nursing competence: a cross-country comparison

**DOI:** 10.1186/s12912-018-0284-0

**Published:** 2018-04-03

**Authors:** Brigid M. Gillespie, Emma B. Harbeck, Karin Falk-Brynhildsen, Ulrica Nilsson, Maria Jaensson

**Affiliations:** 1 School of Nursing & Midwifery, Griffith University, Gold Coast, QLD Australia; 2 Gold Coast Hospital and Health Service, Gold Coast, QLD Australia; 3 National Centre of Research Excellence in Nursing, Griffith University, Gold Coast, QLD Australia; 4 Menzies Health Institute Queensland, Griffith University, Gold Coast, QLD Australia; 5 Faculty of Medicine and Health, School of Health Sciences, Örebro University, Örebro, Sweden

**Keywords:** Perioperative nursing, Competence, Cross-national, Survey, Patient safety

## Abstract

**Background:**

Throughout many countries, professional bodies rely on yearly self-assessment of competence for ongoing registration; therefore, nursing competence is pivotal to safe clinical practice. Our aim was to describe and compare perioperative nurses’ perceptions of competence in four countries, while examining the effect of specialist education and years of experience in the operating room.

**Methods:**

We conducted a secondary analysis of cross-sectional surveys from four countries including; Australia, Canada, Scotland, and Sweden. The 40-item *Perceived Perioperative Competence Scale-Revised* (PPCS-R), was used with a total sample of 768 respondents. We used a factorial design to examine the influence of country, years of experience in the operating room and specialist education on nurses’ reported perceived perioperative competence.

**Results:**

Regardless of country origin, nurses with specialist qualifications reported higher perceived perioperative competence when compared to nurses without specialist education. However, cross-country differences were dependent on nurses’ number of years of experience in the operating room. Nurses from Sweden with 6–10 years of experience in the operating room reported lower perceived perioperative competence when compared to Australian nurses. In comparing nurses with > 10 years of experience, Swedish nurses reported significantly lower perceived perioperative competence when compared to nurses from Australia, Canada and Scotland.

**Conclusion:**

Researchers need to consider educational level and years of experience in the perioperative context when examining constructs such as competence.

## Background

Throughout many countries, professional bodies rely on yearly self-assessment of competence for ongoing registration [1]. Thus the issue of nursing competence is a fundamental aspect of safe clinical practice. Competence is described as a combination of skills, knowledge, attitudes, values and abilities that contribute to effective performance [2]. Assessment of clinical competence is therefore crucial in identifying areas where additional professional development and education are required [3]. Nurses’ perceptions of their competence is an integral component of their professional self-image [4] and may affect role and teamwork performance, job satisfaction, recruitment and retention.

Nurses in countries with distinctly different healthcare systems report similar shortcomings in their work environments associated with the quality of hospital care [5, 6]. Previous research has described differences in nursing practice across countries relative to nurses’ perceptions of professional roles and clinical responsibilities [7–9]. Describing where differences occur across countries is important. Although nurses perform similar roles, there is variation in how the tasks within these roles are operationalised [10]. In addition, government and statutory regulations determine nurses’ scope of practice within these roles [1]. There are also variations in models of care, educational training and certification requirements differs widely among countries [10]. These earlier studies demonstrate the power of international comparisons, cross-cultural research, which offers opportunities to promote international harmonisation in nursing [9, 10].

While previous research has examined differences in nursing practice and healthcare contexts across generalist areas of nursing [11, 12], there is limited research that examines whether such differences exist relative to the specialised area of perioperative practice. In perioperative nursing, these specialisations include nurse anaesthetist, anaesthetic assistant, circulating, instrument, post-anaesthetic care unit roles [10]. Although clinical practices are circumscribed across countries, there is little data to describe cross-country differences relative to self-reported competence in the context of nurse education and years of perioperative experience. Previous work undertaken in the Australian context with samples ranging from 134 to 1178 nurses suggests that education and years of perioperative experience are predictors of perceived competence [13, 14]. However, there have been few, if any, cross-national studies undertaken to describe these relationships. While practice standards may be similar across developed countries, clinical practice and educational preparation is likely to differ from country to country. In this study, we hypothesised that there would be interaction effects between country and years of experience, and country and specialist qualifications, relative to perioperative nurses’ perceived competence.

## Methods

### Design

A secondary analysis of cross-sectional surveys from four countries including; Australia, Canada, Scotland, and Sweden was undertaken using the Perceived Perioperative Competence Scale-Revised (PPSC-R) [15]. The aim of the primary analysis in the Australian context was to refine the PPCS-R to make a more parsimonious scale. The aims of the primary research in the Canadian and Scottish contexts was to compare differences in perceptions of competence among nurses and perioperative technicians. The aim of the primary study undertaken in Sweden was to test the PPSC-R in the Swedish context using confirmatory factor analysis. Demographic data related to gender, years of perioperative experience in the operating room (OR) and specialist qualifications were collected. ‘Specialty education’ is defined as education that is gained beyond that acquired in a baccalaureate or diploma level nursing course [13]. Specialist qualifications included graduate certification qualifications (e.g., CNOR, EBN), diplomas, masters, and professional and research doctorate degrees.

### Participants, settings and sampling

The Australian sample was drawn from 575 RNs practising in the OR departments of 3 large metropolitan hospitals in Queensland. The Canadian sample was drawn from 301 RNs working in the OR departments of 3 large inner city hospitals in Toronto, Ontario. The United Kingdom (UK) sample of 203 perioperative nurses and practitioners was drawn from 3 NHS Trusts in eastern Scotland, spanning from Aberdeen to Larbert. The Australian, Canadian and UK samples were drawn using convenience methods. A census sample of 2902 nurses who were members of the Swedish Association of Health Professionals as either OR nurses or Registered Nurse Anaesthetists (RNA) were emailed independently through the Association. Survey data across samples was collected from 2011 to 2016.

### Measures

Across the four countries, the PPSC-R [15] was used to measure perceived competence. The iterative development and validation of the 40-item PPCS-R is based on a series of earlier qualitative and quantitative studies [3, 13, 15–18]. The PPCS-R uses a 5-point Likert response option, with scores ranging between 40 to 200 and higher scores indicating greater levels of reported perceived competence [15]. The PPCS-R comprises 6 factors that indicate different dimensions of perioperative competence*: Foundational knowledge and skills; Leadership; Collaboration; Proficiency; Empathy; and Professional development* [15]. The total PPSC-R score is based on the sum of all 40 items in each of the 6 factors.

During testing, the PPCS-R demonstrated robust psychometric properties in census sample of Australian perioperative nurses (*n* = 1122). In this sample, the results of the final exploratory factor analysis (EFA) with the 6 factors accounted for 58.3% of the total variance, and Cronbach’s alpha (α) ranged from .81 to .89 [15]. The PPSC-R has been used in the Australian and Canadian contexts, and the internal consistency (α) for the PPCS–R was .96 and .97 respectively [9, 19]. In the current study, the PPCS-R was used as the dependant variable for the analyses. More recently, the PPSC-R was translated into Swedish and validated in a national census of 2, 902 nurses who practised in circulating/instrument and nurse anaesthetist roles [20]. Confirmatory factor analysis (CFA) supported the factor structure of the PPSC-R using the six latent factors and indicated an acceptable model fit for the Swedish sample.

### Statistical analysis

IBM SPSS Statistics version 22 was used to analyses the data. Descriptive and inferential analyses were used. Descriptive statistics were used to measure variable dispersion across the sample. The types of analyses used were determined by the level of the data (i.e., categorical or continuous) and its distribution. Respondents’ composite PPCS–R and subscale scores (for the six domains) were measured as continuous variables while gender, primary role, specialty qualifications and years of OR experience were analysed as categorical variables. Cronbach’s alpha (α) was used to determine the internal consistency of the PPCS–R.

Inferential analyses included 4 (country: Australia, Canada, Scotland and Sweden) × 3 (years of experience in the OR: 0–5 years, 6–10 years and > 10 years) × 2 (specialist and non-specialist qualification) factorial Analysis of Variance (ANOVA). We used an alpha level of 0.05 to determine statistical significance. Partial eta squared (*η*_p_^2^) was used as an indication of effect size. Traditionally *η*_p_^2^ values of 0.01, 0.06 and 0.14 represent small, medium and large effect sizes [21]. Levene’s test for homogeneity of variances was used to assess equality of variances across groups. When this assumption was non-significant (*p* > .05), equal variances were assumed and a Bonferroni correction was used to assess significance for multiple comparisons. When homogeneity was violated *p* < .05, and equal variances were not assumed and the Games-Howell correction was used to assess significance.

## Results

Survey data across the four countries was collected from 2011 to 2015. Response rates were as follows; Australia 30.6% (176/575), Canada 76.5% (134/175), Scotland 71.1% (214/301), and Sweden 38.5% (1033/2679). Across the four countries, the combined sample of 1557 cases was reduced to a final sample of 768. The Swedish sample had 1033 cases of data and this was disproportionate to the other 3 samples sizes. Thus we used a stratified random sample of 250 cases was selected from the original sample. The 250 cases were stratified by nursing role, resulting in 125 OR nurses and 125 RNAs. However, across the samples 6 cases were also removed due to missing data. The sample demographics for the 4 countries are provided in Table 1. Internal consistency was examined for the current sample with Cronbach’s alpha ranging from 0.94 for Sweden, 0.96 for Scotland and 0.97 for both Australian and Canadian samples.

**Table 1:** Cross country sample demographic results

	Australia	Canada	Scotland	Sweden	Total Sample
*n* (%)	175 (22.8)	132 (17.2)	212 (27.6)	249 (32.4)	768
Gender					
Male	21 (12.0)	14 (10.6)	24 (11.3)	38(15.3)	97 (12.6)
Female	154 (88.0)	118 (89.4)	188 (88.7)	211 (84.7)	671 (87.4)
Nursing role					
EN	0 (0.0)	14 (10.6)	4 (1.9)	0 (0.0)	18 (2.3)
RN/OR practitioner	131 (74.9)	118 (89.4)	192 (90.6)	0 (0.0)	441 (57.4)
Clinical nurse/manager	44 (25.1)	0 (0.0)	16 (7.5)	0 (0.0)	60 (7.8)
RNA	0 (0.0)	0 (0.0)	0 (0.0)	125 (50.2)	125 (16.3)
OR nurse	0 (0.0)	0 (0.0)	0 (0.0)	124 (49.8)	124 (16.2)
Specialist qualification					
Yes	51 (29.1)	103 (78.0)	96 (45.3)	58 (23.3)	460 (59.9)
No	124 (70.9)	29 (22.0)	116 (54.7)	191 (76.7)	308 (40.1)
Years in the OR					
0–5 years	64 (36.6)	38 (28.8)	59 (27.8)	50 (20.1)	211 (27.5)
6–10 years	33 (18.9)	26 (19.7)	42 (19.8)	60 (24.1)	161 (21.0)
> 10 years	78 (44.5)	68 (51.5)	111 (52.4)	139 (55.8)	396 (51.5)

*Abbreviations: EN* = enrolled nurse, *RN* = registered nurse, *RNA* = registered nurse anaesthetist, *OR* nurse = operating room (i.e., circulating/instrument/anaesthetic roles)

Descriptive results of total PPCS-R scores for each country relative to years of OR experience and specialist qualification are detailed in Table 2. There was a significant main effect of specialist education *F* (1,706) = 4.0, *p =* .047 (η_p_^2^ = .01). A main effect is the effect of one the independent variable i.e., specialist education on the dependent variable PPC-R, ignoring all other independent variables. Overall, respondents who had specialist qualifications reported higher PPC (*M* = 163.1, *Std error* = 1.3, 95% CI 160.6–165.6) than those without specialist education (*M* = 159.5, *Std error* = 1.3, 95% CI 156.9–162.0).

**Table 2:** PPSC-R scores across countries relative to specialist education and years of experience in the OR

Country	Variable	Mean	Std. error	95% CI
Australia				
	Specialist qualification			
	Yes	166.2	3.3	159.7–172.6
	No	161.4	1.9	157.7–165.1
	Years of experience in the OR			
	0-5 years	148.1	4.0	140.2–156.0
	6–10 years	170.1	3.3	163.6–176.6
	> 10 years	173.1	2.2	168.8–177.5
Canada				
	Specialist qualification			
	Yes	166.3	2.1	162.1–170.5
	No	157.9	4.0	150.0–165.9
	Years of experience in the OR			
	0-5 years	144.3	4.9	134.6–154.0
	6–10 years	166.9	3.9	159.2–174.6
	> 10 years	175.2	2.7	170.0–180.5
Scotland				
	Specialist qualification			
	Yes	164.9	2.0	160.9–168.8
	No	163.3	2.2	159.0–167.6
	Years of experience in the OR			
	0-5 years	152.0	2.6	146.9–157.1
	6–10 years	163.5	3.1	157.5–169.5
	> 10 years	176.7	2.0	172.8–180.6
Sweden				
	Specialist qualification			
	Yes	156.3	2.8	150.9–161.8
	No	154.7	2.0	150.8–158.5
	Years of experience in the OR			
	0-5 years	144.9	2.7	139.5–150.2
	6–10 years	162.2	3.0	156.3–168.0
	> 10 years	158.7	2.6	153.5–163.8

**Fig. 1:**
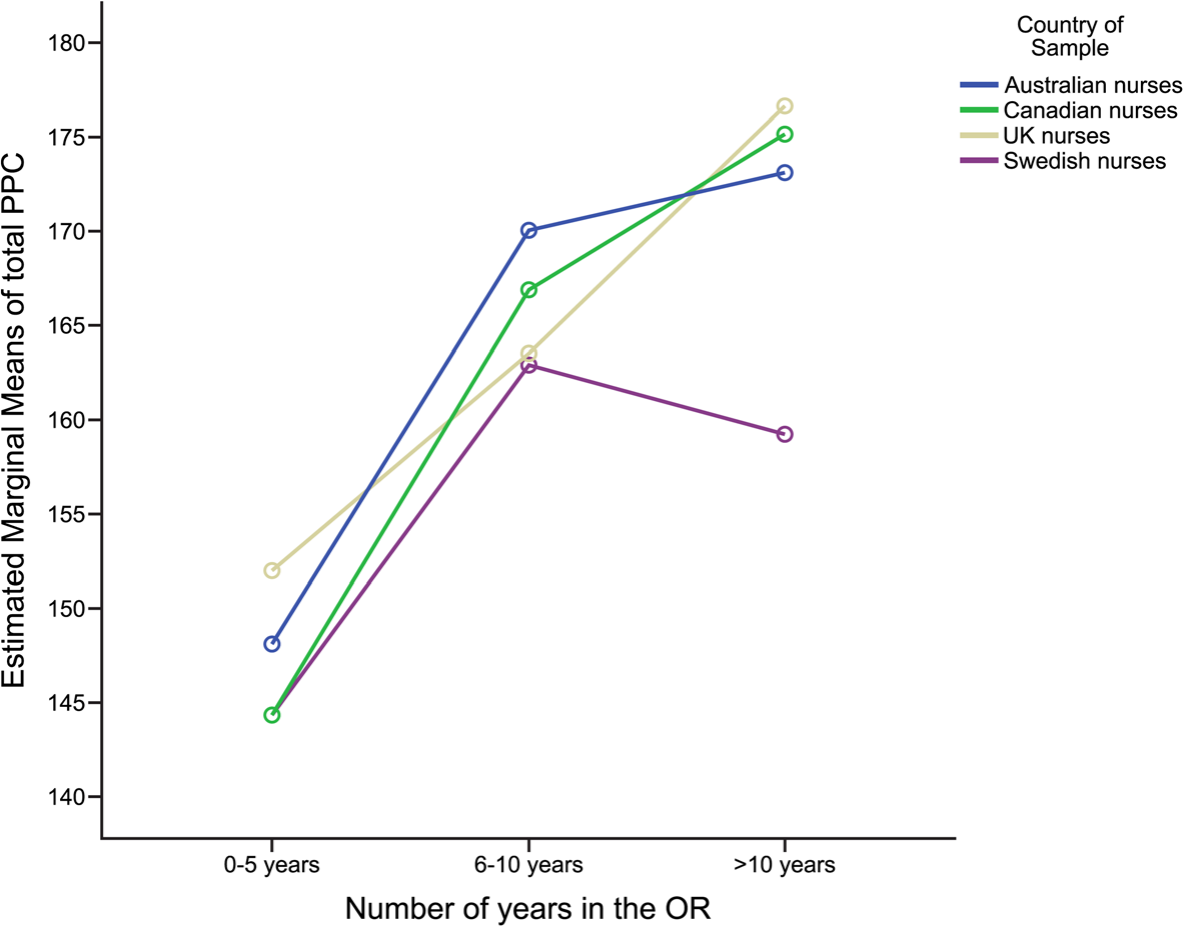
Interaction plot based on s PPSC-R scores showing main effects for country and years of OR experience

There were also significant main effects for Country *F*(3,706) = 6.5, *p <* .001 (η_p_^2^ = .03), and years of OR experience *F*(2,706) = 58.5, *p <* .001 (*η*_p_^2^ = .14). However, these were qualified by a significant interaction between these variables *F*(6,706) = 2.5, *p =* .022 (η_p_^2^ = .02), as shown in Fig. 1. This interaction effect represents the combined effects of independent variables i.e., country and years of OR experience, on the dependent variable, PPC-R. Post hoc multiple comparisons were run by splitting analyses via years of experience in the OR (0–5 years, 6–10 years and > 10 years), which resulted in three one-way ANOVAs with country as the independent variable. There were no significant country differences in respondents with 0–5 years of OR experience *F*(3,193) = 1.23, *p =* .300. However, country differences did occur in total reported PPC for respondents with 6–10 years of experience *F*(3,152) = 4.33, *p =* .006 (η_p_^2^ = .08) and those with > 10 years of experience in the OR, *F*(3,373) = 14.9, *p <* .001 (η_p_^2^ = .11).

Pairwise comparisons between the countries for respondents with 6–10 years of experience in the OR show that in general, Australian nurses reported higher PPC when compared to the Swedish nurses only, *t* = − 3.6(91), *p* = .001. There were no significant differences between nurses in any other countries. For those with > 10 years of experience, nurses from Sweden reported significantly lower PPC when compared to nurses from Australia *t* = − 3.7(211), *p* < .001, Canada *t* = − 5.8 (162.9), *p* < .001 and UK *t* = − 5.2(236), *p* < .001 respectively. No other significant differences were observed, between nurses with > 10 years’ experience, in reported PPC. Means, standard error and 95% confidence intervals are shown in Table 2.

## Discussion

In this secondary analysis of cross-national data, we examined the influence of specialist education and years of experience across a four-country sample in reported nurses perceived perioperative competence (PPC). Across the combined sample, 54% of survey respondents had over a decade of perioperative experience. Concomitantly, respondents reporting greater levels of PPC had more years of perioperative experience; thus the effect of years of experience follows the expected trend [13, 15, 22–24], with the exception of Sweden and to a lesser extent, Australia. In this study, 29% and 23% of Australian and Swedish nurses respectively reported having specialist qualifications. Our findings also suggest a decline in PPC for nurses from Sweden and Australia who had 6–10 years of perioperative experience and for nurses with 10 years or more perioperative experience; nurses from Sweden reporting lower PPC when compared to the other country samples. We suggest that these differences in self-reported competence may also be attributed to cultural interpretation. It may be that humility, self-control and frugality are more highly valued traits in Swedish culture. The Swedish word “lagom” meaning *less is more, just enough*, describes the basis of Swedish national psyche which is characterised by consensus, equity, and using a balanced and moderate approach [25]. Thus, we recommend caution in interpretation of these results as the lower levels of PPC among the Swedish sample are not necessarily indicative of diminished clinical mastery, performance or achievement.

Across the entire cross-national sample in this study, nearly 60% of perioperative nurses reported having a specialist qualification, with the highest numbers of nurses from Canada (78%) and Scotland (45%). Our findings suggest that there was an effect of specialist education, where nurses regardless of country of origin with specialist qualifications, reported higher levels of PPC when compared to nurses with undergraduate or vocational qualifications. Similar results have been described in a litany of earlier research on the influence of specialist or specialty education relative to self-reported competence [13, 15, 23, 26, 27]. Undoubtedly, there is likely diversity of educational programs offered at both the undergraduate and specialist levels among the cross-national samples in this study; thus, direct comparisons may be difficult to draw. For instance, in Australia, there is a range of postgraduate programs available to perioperative nurses, while in Canada, perioperative nurses are required to write a perioperative certification exam. In the UK, there are limited opportunities for nurses to extend their skills formally, while in Sweden, perioperative nurses are required to undertake postgraduate studies. Our results suggest the increases in PPC were consistent across all groups. Seminal cross-national and multisite research [5, 6, 28] confirms a logical connection between the level of nursing education, clinical judgement and patient outcomes.

There may be other reasons, not accounted for in our study to explain cross-country differences noted herein. First, while role may contribute significantly to clinical performance, it is unclear how or why it may affect perceived perioperative competence. In our study, small sample sizes and the differences in role identification across countries precluded analysis based on role categories. Second, the likely cultural differences in the conceptual interpretation of individual words or statements in the scale may have indirectly influenced our results. Finally, organisational macro-management strategies based on professional rewards may have a positive impact on nurse competence. For instance, competency-based management approaches that accurately assess a nurse’s performance, skills and abilities have the potential to increase nurses’ understanding of their strengths and weaknesses [29]. Such approaches have led to increases in nurses’ engagement with learning and the pursuit of additional qualifications that increase their expertise. The PPCS-R scale has potential to be used by front-line perioperative nurses as a tool to stimulate reflection on their clinical practice. This information can be used to assist perioperative nurse educators in tailoring their professional development programs to address strengths and weaknesses identified by front-line nurses.

### Limitations and strengths

Although the PPC-R has been psychometrically evaluated in English [15] and Swedish [20], and has demonstrated robust properties, we acknowledge some limitations. First, most samples were drawn from convenience samples and only one sample from Sweden was population-based. Second, the response rates across all countries and samples ranged from 30% to 76%, while reasonable, may be difficult to generalise beyond the immediate sample. Third, the differences in the PPC-R sum scores between countries may reflect methodological differences between studies, such as when the conditions of data collection differed (surveyed at hospital versus emailed). Additionally as no random samples were drawn, differences may also be due to a population bias. This is one of the most frequent and most problematic shortcomings in cross-cultural studies [30]. Fourth, using years of experience as a categorical variable with pre-defined groupings is common in the literature; however this can result in reduced statistical power and increased risk of type two error [31]. Due to the secondary data analysis design of the current sample, questionnaire data used for the sample only provided experience as a categorical variable for examination. As we found an effect of years of experience, future research should examine the how much variance can be explained by years of experience as a continuous variable. Fifth, these results were based on secondary analyses of the primary data collected several years earlier, and may not account for changes over time. Finally, the results of this cross-country comparison are based on self-assessment rather than on observed behaviours. Yet, self-reported measures are cost effective and provide opportunity for self-reflection.

## Conclusion

To our knowledge, this is the first study to describe cross-country comparisons relative to perioperative nurses’ perceived competence based on country, years of experience and specialty education. Our results also suggest that researchers need to take into account education qualifications and years of experience in the perioperative context when examining constructs such as competence. Cross-country comparative research contributes significantly to the body of knowledge about a specific country as well as providing information on important factors researchers need to consider when evaluating eclectic constructs such as competence in nursing samples. As such, our results may contribute to the examination of population norms for researchers who may want to evaluate perceived competence.
